# High Prevalence of Cefotaxime Resistant Bacteria in Grazing Beef Cattle: A Cross Sectional Study

**DOI:** 10.3389/fmicb.2019.00176

**Published:** 2019-02-07

**Authors:** Sarah Markland, Thomas A. Weppelmann, Zhengxin Ma, Shinyoung Lee, Raies A. Mir, Lin Teng, Amber Ginn, Choonghee Lee, Maria Ukhanova, Sebastian Galindo, Chad Carr, Nicolas DiLorenzo, Soohyoun Ahn, Jae-Hyung Mah, Hae-Yeong Kim, Volker Mai, Ray Mobley, J. Glenn Morris, KwangCheol Casey Jeong

**Affiliations:** ^1^Department of Animal Sciences, University of Florida, Gainesville, FL, United States; ^2^Emerging Pathogens Institute, University of Florida, Gainesville, FL, United States; ^3^Herbert Wertheim College of Medicine, Florida International University, Miami, FL, United States; ^4^Department of Agricultural Education and Communication, University of Florida, Gainesville, FL, United States; ^5^North Florida Research and Education Center, University of Florida, Marianna, FL, United States; ^6^Department of Food Science and Human Nutrition, University of Florida, Gainesville, FL, United States; ^7^Department of Food and Biotechnology, Korea University, Sejong, South Korea; ^8^Institute of Life Sciences and Resources and Department of Food Science and Biotechnology, Kyung Hee University, Yongin, South Korea; ^9^Department of Epidemiology, College of Public Health and Health Professions and College of Medicine, University of Florida, Gainesville, FL, United States; ^10^Department of Animal Science, Florida Agricultural and Mechanical University, Tallahassee, FL, United States; ^11^Department of Medicine, College of Medicine, University of Florida, Gainesville, FL, United States

**Keywords:** cefotaxime, antibiotic resistance, beef cattle, cross sectional study, farm management survey

## Abstract

Although the over-use of antibiotics during food animal production is a potential driver of antimicrobial resistant microorganisms (ARMs), a high prevalence of cefotaxime resistant bacteria (CRB) has been observed in grazing animals raised without antibiotic supplementation. In this cross-sectional study, the prevalence and concentration of CRB in beef cattle on grazing farms were investigated. Fecal samples from the recto-anal junction of cattle (*n* = 840) and environmental samples (*n* = 258) were collected from 17 farms in North and Central Florida in the United States, and a survey of farm characteristics, animal husbandry practices, and antibiotic usage was conducted. CRB were detected in fecal samples from 47.4% of all cattle, with the prevalence ranging from 21.1 to 87.5% on farms, and significantly higher (*P* < 0.001) in calves compared to adult cows (54.1 vs. 41.8%). Environmental samples had a higher prevalence than fecal samples (*P* < 0.001), with CRB detected in 88.6% of water, 98.7% of soil, and 95.7% of forage samples. Compared to the concentration (log CFU/g) of CRB in fecal samples (2.95, 95% CI: 2.89, 3.02), the concentration of CRB was higher (*P* < 0.001) in soil and forage samples (5.37, 95% CI: 5.16, 5.57) and lower (*P* < 0.001) in water samples (1.08, 95% CI: 0.82, 1.36). Soil microbiota from farms with high prevalence of CRB clustered closer together and the proportion of Phylum Proteobacteria was higher on farms with high prevalence of CRB resistance. Large farming operations were associated with a 58% higher likelihood of CRB detection in fecal samples. Regular cleaning of drinking troughs and the addition of ionophores to feed were associated with CRB reduction in fecal samples. Taken together, the widespread of CRB into both cattle seldom treated with cephalosporin antibiotics and the surrounding environment suggests the environment is a natural source of antimicrobial resistance in beef cattle.

## Introduction

The emergence of antimicrobial resistant microorganisms (ARMs) is one of the most critical public health problems in the 21st century (Friedman et al., 2016). Approximately 23,000 people are killed by ARMs each year in the United States (Lutgring et al., 2018). Increased use of antibiotics in health care settings and a lack of development of new antibiotic or alternative compounds, have likely contributed to the rise of drug resistant microbes (Norrby et al., 2005; Turnidge and Christiansen, 2005; Holmes et al., 2016). Likewise, the emergence of ARMs has limited the efficacy of antibiotics and created difficulties in controlling bacterial infections (Kollef and Fraser, 2001).

Recently, extended-spectrum β-lactamase (ESBL)-producing *Enterobacteriaceae* have become a major concern. In 2017, the World Health Organization (WHO) identified that ESBL-producing *Enterobacteriaceae* as one of the priority pathogens included in a catalog of 12 families of bacteria (Bulabula et al., 2017). Since ESBLs exert resistance to the majority of β-lactam antibiotics including penicillins, cephalosporins, and monobactams, most of the ESBL-producing bacteria are resistant to multiple antibiotics (Rupp and Fey, 2003). Especially, cephalosporins are widely used in human medicine, and ESBL-producing bacteria were initially identified primarily in human clinical settings (Geser et al., 2012; Seiffert et al., 2013). However, the ESBL-producing microorganisms have been increasingly isolated in animals (Fischer et al., 2017; Michael et al., 2017), and it has been demonstrated that these microorganisms are being transmitted among human and animal populations, with documented and frequent entry into the human food chain (Carattoli, 2008; Schaufler et al., 2016; Mukerji et al., 2017).

In North America, there are different types of cattle operation systems such as grazing cow/calf or feedlots for production of mature animals. Due to high concentrations of animals, feedlot cattle are more frequently treated with antibiotics to prevent and/or cure animal diseases and thus it could be a critical point to acquire ARMs. Thus, most studies have focused on feedlot cattle for the occurrence of ARMs (Alexander et al., 2008; Noyes et al., 2016a,b), but less attention has given to grazing cow/calf operations because of lack of extensive usage of antibiotics. In addition to antibiotic usage, factors such as farmers working on cattle (Dierikx et al., 2013; Hammerum et al., 2014), duration of manure storage (Pruden et al., 2013), animal age (Mir et al., 2018), and seasonality (Mir et al., 2018) also affect the prevalence of ARMs on farms.

We have recently reported that the prevalence of cefotaxime resistant bacteria (CRB) in beef cattle housed and raised at University’s research facility without cephalosporin antibiotics is high, especially in young calves (Mir et al., 2016, 2018). These findings suggest that not only antibiotic use but also environmental factors such as soil, plants and water may be critical sources for ARMs in grazing cattle, therefore in this paper we analyzed antibiotic use and other factors such as forage, water, soil, and farm management on the prevalence of CRB in 17 farms located in Florida, United States to understand the magnitude of these factors on the prevalence of ARMs in cattle on commercial cow/calf operations.

## Materials and Methods

### Ethics Statement

Standard practices of animal care and use were applied to animals used in this project. The research protocols used in this study were approved by the University of Florida Institutional Animal Care and Use Committee (IACUC Protocol #: 201408629). Protocols and tools for human data collection were approved by the University of Florida Institutional Review Board as “Exempt” (IRB# 15U0896).

### Study Locations and Enrollment

**FIGURE 1:**
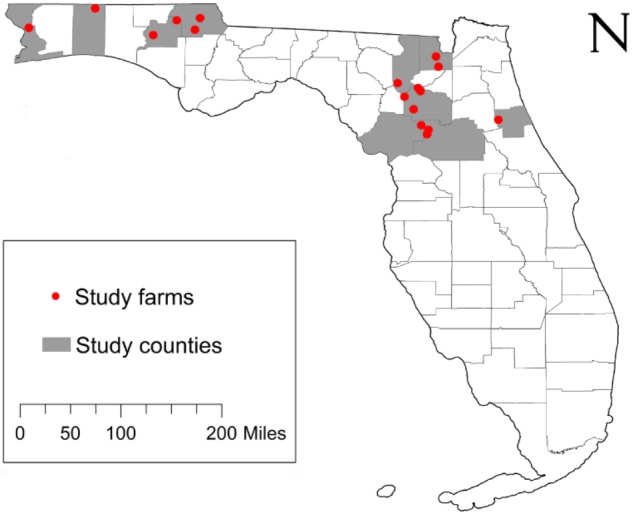
Study location and sample origin. The study enrollment locations included 17 farms from 9 counties across North and Central Florida, United States. The farms appear (red circles) relative the state and county boundaries of Florida.

The original design of this study included sampling approximately 50 beef cattle and 10 environmental samples from 20 farms across nine counties in North and Central Florida. Commercial beef cattle farms were identified using the county extension agents for the University of Florida’s Institute of Food and Agricultural Sciences (IFAS) and 20 farms were randomly selected for study enrollment. Of the 20 farms identified, two farms did not consent to participate and one farm was not able to be contacted (85% response rate). Access to the farms to collect fecal samples was permitted by farm owners. Between February and June 2016, fecal and environmental samples were collected from the 17 participating farms (Figure 1 and Table 1). Additionally, a survey questionnaire (Dillman et al., 2014) of farm characteristics, management practices, farm hygiene, biosecurity, and antibiotic usage was administered to each farm during sample collection. The survey questionnaire was developed by the project’s evaluator in collaboration with a panel of experts that validated the accuracy and relevance of both question stems and response options. The resulting instrument was directly administered to the farm representative interacting with the research team during sample collection. At each farm, a member of the research team conducted an assisted one-on-one interview with the farm representative to complete the survey. The survey responses were entered in Qualtrics, an online survey management system, to generate initial responses. Survey data were further analyzed using the Statistical Package for the Social Sciences (SPSS) and following appropriate quantitative methods for this type of data (Lipsey and Cordray, 2000).

**Table 1:** Farm identification and sample collection.

Enrollment locations		Fecal and environmental sample sizes						
Farm	County	Calves	Cows	Fecal total	Forage	Soil	Water	Total
1	Washington	18	20	38	10	5	10	63
2	Okaloosa	11	31	42	2	2	0	46
3	Jackson	40	43	83	11	5	5	104
4	Washington	40	47	87	8	5	10	110
5	Alachua	14	30	44	5	5	5	59
6	Escambia	4	13	17	7	5	5	34
7	Levy	32	42	74	5	5	5	89
8	Levy	30	21	51	5	4	5	65
9	Alachua	31	29	60	5	5	4	74
10	Baker	10	12	22	5	5	5	37
11	Jackson	39	42	81	5	5	10	101
12	Alachua	23	27	50	0	0	0	50
13	Alachua	26	28	54	5	5	5	69
14	Columbia	8	15	23	5	5	5	38
15	Flagler	23	21	44	5	5	5	59
16	Baker	22	24	46	5	6	4	61
17	Alachua	12	12	24	5	5	5	39
All farms		383	457	840	93	77	88	1,098


### Animal Management and Sample Collection

Farms selected to be enrolled in the study are representative of typical cow/calf operations in North and Central Florida where animals are kept free-range and low management is required. All farms relied on the use of grazing of warm-season forages such as bermudagrass (*Cynodon dactylon*) or bahiagrass (*Paspalum notatum*) as their main feed resource with the addition of mineral supplementation *ad libitum*. As it is typical in most cow/calf operations in Florida, feed management practices during the winter months include either the use of a concentrate supplement, the use of hay as a form of conserved forage, or the use of winter annual forages for grazing, such as oats (*Avena sativa*), rye (*Secale cereal*), or ryegrass (*Lolium multiflorum*). For the farms sampled, as it is common in cow/calf operations throughout the US, the use of antibiotics was restricted to a therapeutic indication such as systemic or localized infections. Some of the farms enrolled in the study used ionophores, which modify the diversity of bacteria inside the rumen of the cow.

Fecal samples (*n* = 840) were collected from the recto-anal junction (RAJ) of calves (*n* = 383) and cows (*n* = 457) using sterile plastic bags (Whirl-Pak, WI, United States). Environmental samples (*n* = 258) including soil, forage (pasture grass, plants, hay), and water (ponds and watering troughs) were also collected in sterile plastic bags or tubes. The number and type of samples collected from each farm are listed in Table 1. Every sample was transported on ice to the Emerging Pathogens Institute (EPI) in Gainesville, Florida and processed the same day.

### Quantification of Cefotaxime Resistant Bacteria

Upon arrival to the laboratory, each sample was given a unique identification number prior to isolation and quantification of CRB. Two grams of feces was weighed into a sterile 15 mL conical tube and suspended in 5 mL of sterile 30% glycerol; one gram of soil or forage material was suspended in 10 mL of sterile saline (0.85% NaCl); 25 mL of water was centrifuged and re-suspended in 1 mL of sterile saline (0.85% NaCl). From the resulting solutions, 100 μL aliquots of fecal and environmental samples were plated at three dilutions (10^0^, 10^-2^, and 10^-3^), onto MacConkey agar (Becton Dickinson, Sparks, MD, United States) containing 4 μg/mL cefotaxime prior to overnight incubation at 37°C. After 18–24 h, bacterial colonies were enumerated, recorded and stored for future genetic characterization. A total of 3,175 CRB were isolated. To ensure if isolated CRB colonies were truly resistant to cefotaxime antibiotic, minimum inhibitory concentration (MIC) of 87 isolates was measured by broth microdilution according to CLSI guidelines (CLSI, 2015). The 87 isolates were selected at random from different farms and environmental sample types; all tested isolates were resistant to cefotaxime antibiotic.

### Statistical Analyses of the CRB Prevalence and Concentration

After multiplication by the appropriate dilution factor, the number of CRB was calculated for each sample as colony forming units (CFU) per gram of feces, soil, or forage, and for each mL of water. The number of fecal samples that contained CRB was used to calculate the prevalence (and 95% confidence intervals) by dividing the number of positive samples by the total number of samples in that category (i.e., farm, for cows *vs.* calves, or sample type). For statistical analyses, the average CFU for each farm or sample type was log_10_ transformed to obtain a normalized distribution. Since the concentration of CRB is directly correlated with the prevalence, only the CRB-positive samples were considered in the comparison of CRB concentration between groups. Statistical comparisons of the prevalence and concentration of CRB in different sample types, between cows *versus* calves, or by response to survey items were conducted using simple logistic and linear regression models, respectively. One-way analysis of variance (ANOVA) was used to examine differences in the concentration of CRB between farms or sample types, followed by *post hoc* testing (Bonferroni). Bartlett’s test was used to assure the equality of the variances between groups prior to interpretation of ANOVA results. All statistical analyses for CRB prevalence and concentration were conducted using STATA software package (StataCorp, College Station, TX, United States) with a significance threshold of α = 0.05. The map of sample collection farms was generated from country and state administrative units provided by the US Census Bureau (United States Census Bureau [USCB], 2016) using ArcMap software (ESRI, Redlands, CA, United States).

### Microbiota Analysis

DNA from pooled soil samples was isolated using a modified Qiagen stool DNA extraction protocol with an initial bead beating step as previously described (Mai et al., 2009). DNA was amplified using bar-coded Illumina primers targeting the V1 and V2 region of the 16S rDNA. Samples were pooled by combining equimolar amounts of PCR products for multiplexed sequencing. Amplicons were sequenced using the Illumina MiSeq platform. From the resulting raw data, sequences of low quality (USEARCH quality filter and chimera detection) or with a paired read length less than 290 nucleotides were removed from the analysis. Using a modified UPARSE algorithm, the sequences were clustered into Operational Taxonomic Units (OTUs) at similarity levels of 95 and 98%. A representative sequence from each OTU was annotated through the Greengenes 16S rDNA reference database using a Bayesian RDP classifier (DeSantis et al., 2006).

Core diversity measures, including Chao1, Shannon-Weaver and Simpson as well as UniFrac distances were generated using the QIIME software package and the R package *phyloseq* (Caporaso et al., 2010; McMurdie and Holmes, 2013). We calculated the percent relative abundance of bacterial phyla, by combining OTUs with the same phylum level taxonomic classification into the corresponding phylum group. OTUs annotated as “un-classified” or that are classified only up to the kingdom level were manually aligned using tBLAST (Altschul et al., 1990).

The significance of differences in the proportion of samples with the presence/absence of specific OTUs was calculated using the *z*-test. We did adjust for the multiple analyses that were performed by reducing the significance level to *P* < 0.003.

## Results

### The Prevalence of Cefotaxime Resistant Bacteria in the Fecal and Environmental Samples

**FIGURE 2:**
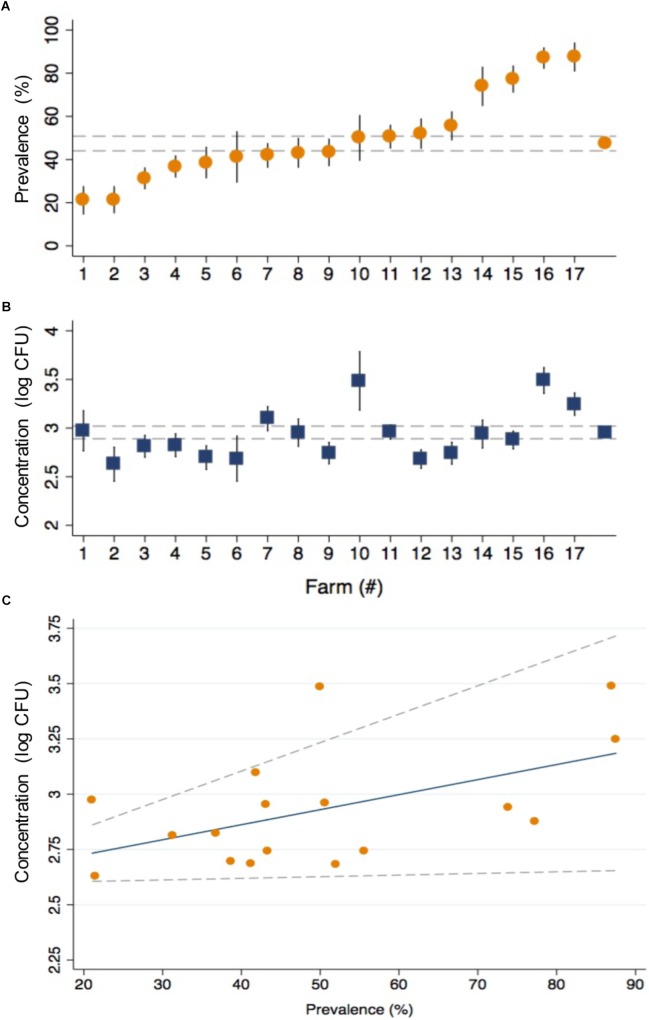
The prevalence and concentration of CRB in beef cattle from 17 cow/calf operations in North and Central Florida. The prevalence **(A)** and concentration (log_10_ CFU) **(B)** of CRB detected in the fecal samples collected from calves and cows are presented with the corresponding standard error (black spike) for the 17 sample collection sites along with the average values from all farms (gray dashed lines). The relationship **(C)** between the concentration (log_10_ CFU) and prevalence of CRB is presented as a scatter plot for the values obtained by farm (orange circles), with the predicted values from a linear regression model (blue line) and 95% confidence intervals (gray dotted lines).

The prevalence of CRB in cattle from all farms was 47.4% and ranged from 21.1 to 87.5% between farms (Figure 2A). The average concentration of CRB (expressed as log_10_ CFU per gram of feces) in cattle that had CRB in their feces (*n* = 398) was 2.95 (95% CI: 2.89, 3.02) and ranged from a minimum value of 2.63 and maximum value of 3.49 between farms (Figure 2B). One-way ANOVA revealed that the average concentration of CRB identified from at least one farm was significantly different (*F* = 4.04, *P* < 0.001) from the others. However only 8 of 136 pairwise comparisons between the average concentration of CRB between farms revealed significant differences; all of which were due to a higher average concentration of CRB from farm 16 ([log_10_ CFU/g] = 3.49, 95% CI: 3.22, 3.76). The prevalence of CRB was significantly higher (*P* < 0.001) in calves compared to cows (54.0 *versus* 41.8%, Table 2). Farms with a higher prevalence of CRB in fecal samples also had an increased concentration of CRB in cattle (*P* = 0.03, Figure 2C), though the prevalence explained only 28% of the variability in the concentration of CRB between farms (*R*^2^ = 0.28).

**Table 2:** Prevalence and concentration of CRB by sample type.

	CRB prevalence			CRB concentration (log CFU)			
		
Sample Types	(*n*)	(%)	95% conf. interval		Average	95% conf. interval	
Cow	457	41.8	37.3	46.3	2.93	2.83	3.03
Calf	383	54.0	49.1	59.0	2.97	2.89	3.06
Fecal	840	47.4	44.0	50.8	2.95	2.89	3.02
Forage	93	95.7	91.6	99.8	5.66	5.43	5.90
Soil	77	98.7	96.2	100.0	5.37	5.16	5.57
Water	88	88.6	82.0	95.3	1.08	0.82	1.36


The prevalence of CRB in the environmental samples was significantly higher (*P* < 0.001) than fecal samples (47.4%), where 95.7% of forage samples (95% CI: 91.6, 99.8), 98.7% of soil samples (95% CI: 96.2, 100), and 88.6% of water samples (95% CI: 82.0, 95.3) had detectable CRB (Table 2). The concentrations of CRB in environmental samples were also significantly different from fecal samples (Table 2). Compared to the average concentration of CRB in fecal samples ([log_10_ CFU/g] = 2.95; 95% CI: 2.89, 3.02), environmental samples had the following average concentrations: forage ([log_10_ CFU/g] = 5.66; 95% CI: 5.43, 5.90), soil ([log_10_ CFU/g] = 5.37; 95% CI: 5.16, 5.57), and water ([log_10_ CFU/mL] = 1.08; 95% CI: 0.82, 1.36). Using one-way ANOVA, the mean concentrations between groups were significantly different (*F* = 585.7, *P* < 0.001). Compared to fecal samples, water samples had 1.86 log_10_ CFU lower CRB, soil and forage samples had 2.42 and 2.71 log_10_ CFU higher CRB; compared to water samples, soil and forage samples had 4.28 and 4.57 log_10_ CFU higher CRB; no significant difference (*P* = 0.153) was observed between the average CRB concentration in soil samples compared to forage samples.

### Relationships Between Farm Characteristics and CRB Prevalence

**FIGURE 3:**
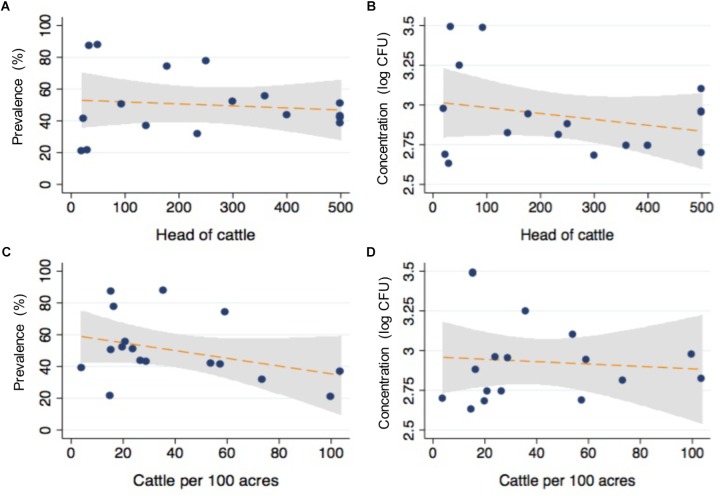
Relationship between cattle density and CRB isolated from commercial beef farms. The relationship between total number of cattle **(A,B)** and the density **(C,D)** of cattle per 100 acres with the prevalence and concentration of CRB from fecal samples are presented as scatter plots from values corresponding to individual farms (blue dots), with the corresponding fits from linear regression models with 95% confidence intervals for the slope (orange line, gray shaded region).

After matching the farm-level survey data with the microbiological findings from individual animals, logistic regression models were conducted to determine the association between survey items and the likelihood of detecting CRB in feces. Selected results are presented in Table 3 with respective sample sizes (n), proportion of animal samples in each survey response item group (%), CRB prevalence (prev %), *P*-values, and odds ratios (OR) with 95% confidence intervals (95% CI). The size of the farms in this study varied between 20 acres and 90,000 acres (median = 1500 acres) with between 20 and 3,600 head of cattle (median = 250); neither of which were associated with the presence of CRB in individual fecal samples (Figure 3, *P* > 0.1 in all cases). Large (>500 cattle) farming operations had higher CRB prevalence than small/medium (<500 cattle) operations (70.1 *versus* 29.9%) and were associated with a 58% higher likelihood of CRB detection in fecal samples (*P* = 0.003, OR = 1.58). No significant associations (*P* > 0.2, in all cases) were observed with the age of the farm operation, the breeds of cattle housed by the farm, the proportion of acres covered by forest, farm topology (rolling hills, flat woods, or improved pasture), geographic location, or county of operation. Participants that frequently reported their soils at “wet or swampy” had a fourfold increase in the likelihood of CRB detection in fecal samples (*P* < 0.001, OR = 4.04) (Data not shown).

**Table 3:** Association between farm characteristics and husbandry and the CRB prevalence.

Farm characteristics	Average	*SD*	Min	Max	OR^a^	*P*-value	95% CI	
Total acres in farm	5930	684	20	90000	0.999	0.248	0.999	1.000
Total number of cattle	578	29	20	3600	0.999	0.119	0.999	1.000

**Survey Items**	**Response**	**(*n*)**	**prop^b^ (%)**	**prev^c^ (%)**	**OR**	***P*-value**	**95% CI**	

Farming type	*Small/medium*	251	29.9	39.4	ref	–	–	–
	*Large*	589	70.1	50.8	1.580	0.003	1.172	2.137

**Animal Husbandry**	**Response**	**(*n*)**	**prop^b^ (%)**	**prev^c^ (%)**	**OR**	***P* value**	**95% CI**	

How many times per month are drinking troughs cleaned?	*Never*	630	75.0	50.2	Ref	–	–	–
	*More than once*	42	5.0	21.4	0.271	0.001	0.128	0.576
	*Once*	87	10.4	36.8	0.578	0.020	0.364	0.918
	*Less than once*	81	9.6	50.6	1.018	0.938	0.641	1.617
Cleaning product used for trough cleaning?	*None*	743	88.5	50.3	Ref	–	–	–
	*Bleach*	97	11.6	24.7	0.324	<0.001	0.200	0.526
Do you supplement ionophores into feed?	*No*	625	74.4	49.4	Ref	–	–	–
	*Yes*	215	25.6	41.4	0.722	0.042	0.528	0.988

**Biosecurity**	**Response**	**(*n*)**	**prop^b^ (%)**	**prev^c^ (%)**	**OR**	***P*-value**	**95% CI**	

Isolation/quarantine for new animals?	*No*	66	7.7	68.2	Ref	–	–	–
	*Yes*	774	92.1	45.6	0.391	<0.001	0.229	0.669
How do you dispose of dead animals?	*Bury*	542	64.5	41.5	Ref	–	–	–
	*Decompose*	298	35.5	58.1	1.940	<0.001	1.464	2.597


*^a^Within each survey item, OR was compared with the first type of response. ^*b*^Proportion of animal samples in each survey response item group (%). ^*c*^Prevalence of CRB in samples linked to survey response item group (%).*

### Relationships Between Animal Husbandry and CRB Prevalence

Most of survey items related to animal husbandry were not significantly associated with the presence of CRB in fecal samples (*P* > 0.4, Table 3), including feed type (grazing, silage, corn or molasses supplemented in the diet), location of supplemental feeders on farm, or reproductive survey items (methods of breeding and acquiring new females). However, the addition of ionophores to feed in 25% of the study animals was associated with a 28% reduction in the likelihood of detecting CRB in fecal samples (*P* = 0.04, OR = 0.72). All farms provided drinking water troughs for animals, though only 25% of farms reported regularly cleaning the drinking troughs. Compared to those who did not clean troughs, cleaning more than once a month was associated with a 73% reduction in the likelihood of detecting CRB (*P* < 0.001, OR = 0.27), cleaning once a month was associated with a 42% reduction (*P* = 0.02, OR = 0.58), and cleaning every two months was no longer associated with a significant reduction (*P* = 0.938, OR = 1.02). Similarly, farmers who reported cleaning animal drinking troughs with bleach (11.6%) had a 68% reduction in the likelihood of detecting CRB (*P* < 0.001, OR = 0.32). Significant predictors of the CRB prevalence also included two biosecurity questions. The use of quarantine or isolation for newly acquired animals from other farms was associated with a 61% lower likelihood of detecting CRB (*P* < 0.001, OR = 0.39); disposal of deceased cattle by decomposition on field was associated with a 94% increase in the likelihood of detecting CRB compared to those that buried deceased animals (*P* < 0.001, OR = 1.94).

### Relationships Between Antibiotic Usage and the CRB Prevalence

Although less antibiotics are used in grazing beef cattle compared to feedlot settings (Marshall and Levy, 2011; Noyes et al., 2016a), the veterinary use of antibiotics is necessary in livestock management and could provide a source of selective pressure for drug resistance. Farmers were surveyed about any past use of antibiotics. The antibiotic usage of each farm is listed in Table 4. None of the farms used antibiotics for growth promotion purpose. Four farms (23.5%) never used antibiotics for bovine disease treatment, 14 farms (82.4%) treated 5% or fewer animals annually, and three farms (17.6%) treated about 10% of the cattle with antibiotics annually. The antibiotics used include oxytetracycline, florfenicol, penicillin, tulathromycin, and ceftiofur for therapeutic purposes. Compared to farms that did not use ceftiofur, the prevalence and concentration of CRB on the three farms that used ceftiofur did not differ significantly (*P* = 0.6 and 0.8). On the majority of farms (81.8%), veterinarians were consulted to diagnose and treat ill or injured animals and no prophylactic use of antibiotics was reported. The antibiotic usage, including total usage and the usage for individual purpose was not correlated with the prevalence or concentration of CRB (*P* = 0.2 and 0.6, respectively).

**Table 4:** Association between farm antibiotic usage and CRB prevalence and concentrations.

Farm	Use	Purpose of antibiotic use			Proportion (%)^a^	Antibiotics used
		Metritis	Foot	Wound		
1^c^	Yes	No	No	No	0	Oxytetracycline and florfenicol
2	Yes	No	Yes	Yes	5	Oxytetracycline, penicillin, and florfenicol
3^c^	Yes	No	Yes	No	10	Oxytetracycline and penicillin
4	Yes	No	No	No	5	Oxytetracycline and tulathromycin
5	Yes	No	Yes	No	5	Oxytetracycline and ceftiofur
6	No	No	No	No	0	Oxytetracycline and tulathromycin
7	Yes	No	Yes	Yes	10	Oxytetracycline and tulathromycin, ceftiofur and florfenicol
8	Yes	No	Yes	No	5	Oxytetracycline, tulathromycin and ceftiofur
9	Yes	No	Yes	Yes	5	Oxytetracycline, tulathromycin, and florfenicol
10	No	No	No	No	0	None
11	Yes	Yes	Yes	Yes	5	Penicillin
12	Yes	No	Yes	Yes	5	Oxytetracycline, tulathromycin, and florfenicol
13	Yes	No	Yes	Yes	5	Oxytetracycline, penicillin, tetracycline, and tulathromycin
14	No	No	No	No	0	None
15	No	No	No	No	0	None
16	Yes	No	Yes	Yes	5	Penicillin
17	Yes	Yes	Yes	Yes	10	Oxytetracycline and tulathromycin

*P*-value (Prev)^b^	0.25	0.17	0.92	0.39		
*P*-value (Conc)	0.69	0.37	0.81	0.83		


*^a^Proportion of cattle that receive drug treatments annually; ^*b*^*P*-value (Prev): the correlation of each parameter and the prevalence of cefotaxime resistant enterobacteria (CRB); *P*-value (Conc): the correlation of each parameter and the concentration of CRB. ^*C*^Farm 1 and 3 use antibiotics only for upper respiratory infection. The frequency Farm 1 uses antibiotics was less than once per year, so the proportion was near 0%.*

### Correlation Between Soil Microflora and Animal Microflora

**FIGURE 4:**
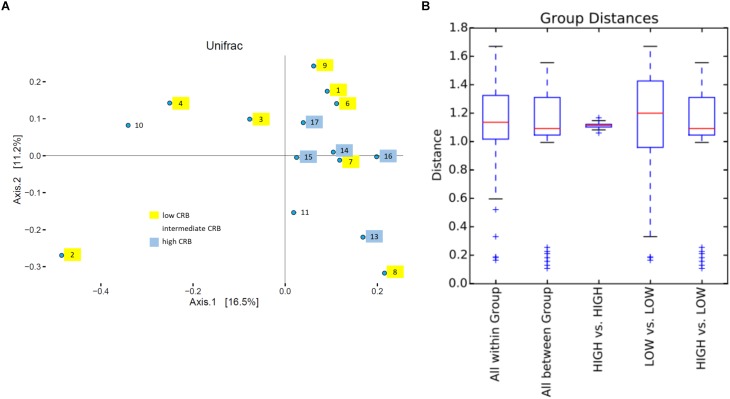
UniFrac beta diversity analysis. **(A)** Principle coordinate plot based on weighted UniFrac. Number and color, respectively indicate farms and corresponding CRB status. **(B)** Box plot of UNIFRACp distances within and between groups.

**FIGURE 5:**
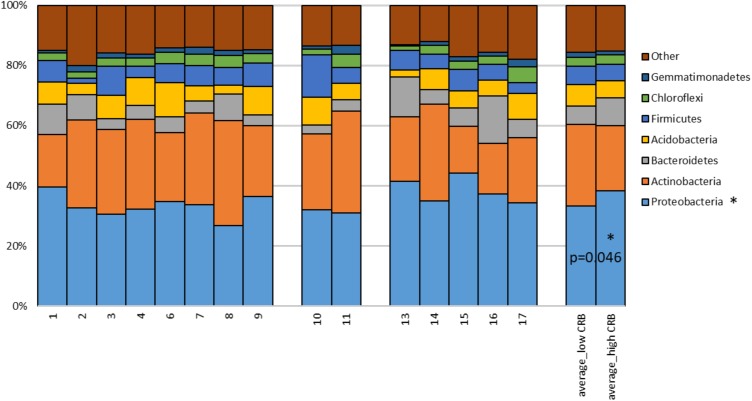
Proportions of 16S rDNA sequences matching to dominant phyla detected in soils. The percentage of reads matching to dominant phyla is provided for each farm soil. Mean proportions of phyla for farms divided by low and high CRB status are provided on the right, *P*-value derived from Student’s *T*-test (unpaired, two-tailed). OTUs matching phyla with sequence abundance < 1.0% (*TM7*, *Planctomycetes*, *Nitrospirae*, *Cyanobacteria*, *WS6*, *Fibrobacteres*, *Armatimonadetes*, *Chlorobi*, *OD1*, *TM6*, *BRC1*, *Elusimicrobia*, *Verrucomicrobia*, *AD3*, *Tenericutes*, *Spirochaetes*, *GN02*, *OP11*, *MVP-21*, *FBP*, *FCPU426, BHI80-139, Kazan-3B-28, GAL15, GN04, GOUTA4, OP3, Fusobacteria, Synergistetes, SBR1093, NC10, SR1, SC4, Lentisphaerae*) and unclassified Bacteria were combined into “other.”

Soil is established as one of the most important sources of ARMs, due to the diversity of microbiota exposed to naturally occurring antibiotics that facilitate selection for resistant genes (Forsberg et al., 2012). We sought to determine if soil microbiota on cow/calf operations are associated with the prevalence of CRB in fecal samples. First, to determine the composition of microbiota in soils, we pooled 5 soil samples collected at different locations from each farm and conducted 16S rRNA sequencing. After removal of low quality and short reads, we retained 1,516,114 sequences with an average of 101,074 reads/sample and a paired read length of 308 nucleotides. 17,991 and 33,164 OTUs were obtained at the 95 and 98% similarity levels respectively. To explore soil microbiota differences between sites with varying degrees of CRB we divided farms into those that had high prevalence of CRB resistance (>55%, *n* = 5) and those with lower prevalence of resistance (<45%, *n* = 8). Farm five soil samples were not included due to technical issues. We did not detect any difference between the two groups in measures of alpha diversity (Supplementary Figure 1). While PCoA plots indicated that samples from farms with high prevalence of CRB clustered closer together (Figure 4A), suggesting that these soil samples shared similar microflora, there was no difference in mean weighted Unifrac distances within *vs.* between groups (Figure 4B). At the phylum level we determined that the proportion of *Proteobacteria* was higher on farms with high prevalence of CRB resistance (38% vs. 33%, *P* < 0.05) (Figure 5). Multiple individual OTUs differed in their prevalence (*P* < 0.05) between farms with high and low prevalence of CRB resistance, (Supplementary Tables S1, S2), suggesting unique OTU patterns correlating with the prevalence of CRB. OTUs classified into *Microbacterium* and *Anaerolinea* genera, *Bacillaceae* and *Sinobacteraceae* families, GKS2-174 and ML635J-21 classes were more abundant in the farms with high prevalence of CRB (*z*- test and two-sided *t*-test *P* < 0.05, Supplementary Table S1) than in the farms with low prevalence. In particular, the farms with high prevalence of CRB had a higher abundance of OTUs belonging to the class Gammaproteobacteria and included the families of *Coxiellaceae*, *Legionellaceae*, and *Sinobacteraceae*. Farms with lower prevalence of CRB had a higher abundance of bacteria from the phylum Proteobacteria including the classes of Alphaproteobacteria and Deltaproteobacteria and the *Acetobacteraceae* family (Supplementary Table S2).

## Discussion

To understand the occurrence of CRB, samples were taken from the RAJ of calves and cows, water, soil, and forage from 17 cow/calf operations. The prevalence and concentration of CRB were investigated to understand factors associated with higher or lower occurrence of CRBs in cow/calf operations. The presence of CRB was detected in nearly half of fecal samples and almost every environmental sample collected, indicating CRB are predominant in beef cattle and in the environment. Likely, the grazing behavior allowed the cattle to have higher contact rates with CRB in soil and forages. In this system, acquisition of ARMs from the environment is probably a critical pathway. In the environment, naturally produced antibiotics are continuously selecting ARMs (Allen et al., 2010), which can explain the high prevalence and concentration of CRB in environmental samples (Table 2). Through soil microflora analysis, we found that the farms with high prevalence of CRB contained in their soil significantly higher relative abundance of Proteobacteria (Figure 5) and specifically OTUs classified into Gammaproteobacteria class (Supplementary Table S1). Gammaproteobacteria represent a major known class of CRB, supporting the notion that the soil environment from these farms could be a source of CRB in these beef cattle (Amos et al., 2014). Furthermore, the concentration of CRB was greater in forage and soil samples than the fecal samples. We speculate that exposure to CRB occurs during grazing/foraging behavior where cattle indirectly ingest soil or ingest plants/forage containing high concentrations of CRB. After ingestion, CRB travel through the digestive tract, leading to the potential for colonization of the RAJ of the animals. This is supported by Hartmann et al. (2012), who found that the environmental and cattle fecal CTX-M positive strains were clonally related. Since CRB were detected in drinking water sources, albeit at lower concentrations than soil or forage (Table 2), this decrease in prevalence could indicate that drinking troughs were also a source of CRB exposure in beef cattle.

It has been proposed that individual animal become colonized with CRB and spread the resistant bacteria throughout the herd via contaminated feces, soils, and forages, which means that increasing animal density would result in a higher herd prevalence and soil concentration of CRB (Liu et al., 2016; Mir et al., 2018). However, as shown in Figure 3, neither the number of animals nor their density was significantly associated with the prevalence and concentration of CRB in cattle, with no differences in the prevalence or concentration of CRB in soil samples. This suggests that animal-to-animal transmission is not the main source of acquiring resistance by animals. It was reported that in feedlots with sub-therapeutic antimicrobial administration, animals shed ARMs with similar genotypes, showing the animal-to-animal strain transmission (Sharma et al., 2008). However, in the cow/calf operations, antibiotic administration occurs less frequently, with antibiotics administered only for disease prevention and clinical treatment purposes, thus reducing the opportunity for selection of ARMs in the gastrointestinal tract and transmission to other animals.

Although the exposure and acquisition of ARMs from the environment by cattle was likely natural, good farming management is necessary to decrease the prevalence and concentration of ARMs (Snow et al., 2012). While all of the study farms allow cattle to graze on forage, farms that supplemented feed with ionophores had an 8% lower prevalence of CRB in their animals compared to farms that did not (Table 3). We speculate that this is due to a reduction in the grazing intake of cattle that is associated with ionophores leading to lower contact rates with CRB in soil and forage (Butaye et al., 2003). Alternatively, ionophores may inhibit growth of CRB directly or indirectly. Controlled study will be needed to clearly understand ionophores effect on the CRB prevalence. Another potential critical control point was identified by the association between cleaning of drinking water troughs and CRB prevalence. Compared with the farms that did not clean water troughs, CRB prevalence was lowest when cleaning took place more than once every month, lower when done once a month, with no effect observed at cleaning frequencies of 2 months or longer. Commercial beef farms in the study, are both bred their own animals and obtained new cows/calves from outside sources, the latter of which could lead to the introduction of bacteria or pathogens that have acquired drug resistance from other geographic locations. This might explain the lower prevalence of CRB on farms with quarantine programs and/or isolation wards for sick animals where antibiotics and antiparasitics are administered followed by observation of animals for 30 to 60 days prior to incorporation of new animals into the herd (Adler et al., 2015; Worthing et al., 2018). Similarly, the reduction in the likelihood of detection of CRB in cattle on farms that buried or burned deceased cattle instead of allowing them to decay in the open, could have resulted from decreased interactions between bacteria, protozoa, and fungal organisms in the soil directly under a decomposing animal that promote the development of resistance factors.

Another study investigated cefotaxime-resistant *E. coli* in dairy and beef cattle farms located in Germany and the association between farm management factors and the prevalence of cefotaxime-resistant *E. coli* (Hille et al., 2017). Out of 60 recruited beef cattle farms, 42 beef cattle units contained cefotaxime-resistant *E. coli* (70%) and the prevalence of cefotaxime-resistant *E. coli* in fecal samples was 41% (112/275). The prevalence detected in that study were lower than the current study, however, some of the same patterns were observed where the prevalence of cefotaxime-resistant *E. coli* was lower in less intensive farming systems with better hygiene. Thus, improved hygiene management (i.e. control of flies, and presence of sick pens) could mitigate the occurrence of resistance in cattle farms.

In the studied farms, the most reported used antibiotic was oxytetracycline (Table 4), a broad-spectrum tetracycline that is commonly applied to feed to prevent diseases and infections. Oxytetracycline and chlortetracycline also count for 42.2% of antibiotic sales for livestock industry, which is the biggest portion, but the tetracyclines only serve as a very small proportion of human medicine industry, counting for 3.9% (Call et al., 2013). The other antibiotics reported by the farmers include florfenicol, penicillin, tulathromycin, and ceftiofur. Florfenicol was commonly used to treat keratoconjuctivitis caused by *Moraxella bovis* (Dueger et al., 1999), bacterial pneumonia and associated respiratory infections (Shin et al., 2005), and infectious pododermatitis (Kehrenberg and Schwarz, 2006). Penicillin was applied to treat multiple bacterial infectious disease in cattle, including metritis (Richarson, 1993). Tulathromycin was widely used to treat bovine respiratory disease, infectious bovine keratoconjuctivitis, and interdigital necrobacillosis (Villarino et al., 2013). Ceftiofur was commonly used to treat metritis and mastitis. Use of all of these antibiotics was limited (<10%), therefore, unlike in feedlots (Sharma et al., 2008), the selective effect of the antibiotics in cow/calf operations was not high. Besides farm management, other environmental factors can also affect the transmission of CRB. For example, runoff and flooding water can have high numbers of ARM, including multidrug-resistant *E. coli* (Blaak et al., 2015; Kawecki et al., 2017), and sewage water may be a reservoir of both ARM and plasmid mediated antibiotic resistant genes (Osinska et al., 2016). These ARM from water sources may be transmitted into cattle ranches during raining or flooding events. Therefore, future studies will be needed to understand the effects of these environmental factors on the prevalence of CRB in cow/calf operations.

To study the potential for natural reservoirs of ARMs, farms that practiced dietary supplementation for growth purposes or other non-therapeutic use of antibiotics were excluded from enrollment. Thus, the study design was not able to compare farms that use antibiotics frequently to those that do not use antibiotics. Likewise, because we focused on bacteria resistant to only one cephalosporin antibiotic, we are unable to speculate on the prevalence and concentration of bacteria resistant to other classes of antibiotics. Since only farms in North and Central Florida were included, the results found in this study might not be representative of beef cattle farms in other regions of Florida, or other states and countries. The inclusion of only farms that agreed to participate in the study could have resulted in selection bias. The survey respondent’s answers to questions regarding animal husbandry practices and use of antibiotics could have also led to misclassification bias. These limitations aside, we believe the identification of a high prevalence of CRB on farms in North Florida is an important finding that warrants further investigations with more rigorous study designs including genetic characterization of bacterial isolates and longitudinal monitoring of CRB colonization in beef cattle.

During this study, CRB were isolated from nearly half of all fecal samples and almost all soil/forage samples collected from 17 beef cattle farms in North and Central Florida. Given the high concentration of CRB in the soil and the seldom use of antibiotics by the farms enrolled in this study, we speculate that the environment is most likely the source of these ARMs and the selective pressures that facilitate their evolution. The ubiquitous nature of drug resistant microorganisms in the soil of these farms and isolation in food animals is of great public health concerns and warrants further investigation to better characterize the natural antibiotic resistome.

## Author Contributions

KJ, SM, TW, ZM, and SL designed the study. SM, TW, ZM, SL, RAM, LT, AG, CL, MU, SG, CC, ND, SA, J-HM, H-YK, VM, RM, JM, and KJ collected and analyzed the data. SM, TW, ZM, SL, RAM, LT, AG, CL, MU, SG, CC, ND, SA, J-HM, H-YK, VM, RM, JM, and KJ drafted the manuscript. SM, TW, ZM, SL, and KJ finalized this manuscript.

## Conflict of Interest Statement

The authors declare that the research was conducted in the absence of any commercial or financial relationships that could be construed as a potential conflict of interest.
