# Supplementary figures and images for: High Prevalence of Cefotaxime Resistant Bacteria in Grazing Beef Cattle: A Cross Sectional Study
Source: Front Microbiol. 2019 Feb 7;10:176. doi: 10.3389/fmicb.2019.00176 (PMC6374349; doi:10.3389/fmicb.2019.00176)

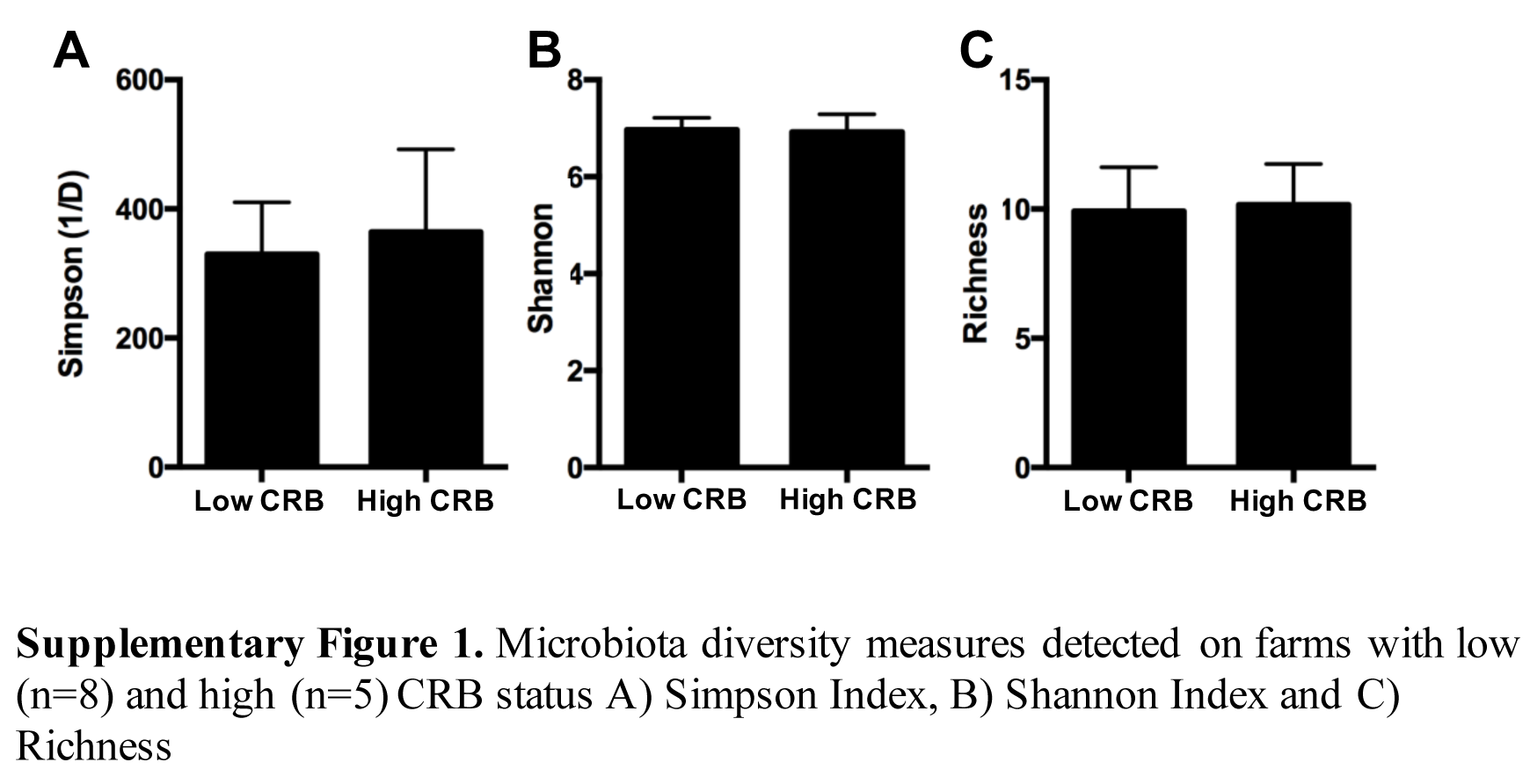

Supplement: Supplementary file 3 [file Image_1.TIF]
